# Clinical Lecture

**Published:** 1881-03

**Authors:** Samuel D. Gross

**Affiliations:** Phila.


### Article II.

A Clinical Lecture. By Samuel D. Gross, m.d., of Phila. Specially reported for the Chicago Medical Journal and Examiner.

Internal Piles.— This is a trouble for which you will be very frequently consulted. You notice this little tumor on the verge of the anus. It is characteristic in its appearance, and is the cause of great pain. The man first noticed its appearance yesterday afternoon, following a passage accompanied by a good deal of straining. The tumor is uncommonly large for a pile. It is of the usual bluish color, and imparts a decided sense of tightness to the touch. Hemorrhoidal tumors are of two kinds, external and internal. The internal pile is within the sphincter ani muscle and consists of a knot of hypertrophied arteries and veins. It is commonly soft and spongy in texture. The external hemorrhoid is of a very different character. It is external to the sphincter ani muscle, but it is very often strangulated by the contraction of that muscle. It consists of an extravasation of blood from the hemorrhoidal vessels, is, in fact a sort of apoplexy at the verge of the anus. As regards the treatment of an external hemorrhoid, Erichsen of London, and Bryant of Grey's Hospital, advise its immediate removal with a knife. This is a truly villainous practice, and attended with great risk of obstinate hæmorrhage. The American surgeon incises the tumor with a bistoury and presses out its contents—*i. e.*, the contained clot of blood. The structure of an external hemorrhoid consists entirely of this clot of blood. The slight operation relieves the pain and tension at once. As an after-treatment the parts should be well bathed with cold water and some medicine given to act on the liver and bowels.

Malignant Disease of the Lip.—This man is a farmer and 55 years of age. He always enjoyed good health until April last, when following a slight local irritation in shaving, a small wart made its appearance on the outer portion of the upper lip.

This wart gradually increased in size. The man consulted a neighboring physician, who laid the wart open and cauterized it every day for a month, with the result you now see,—a roundish, lobulated ulcer, giving forth a sanious discharge and causing frequent twinges of sharp lancinating pain. This ulcer extends from within one-half inch of the median line of the upper lip across the cheek bone, and one and a quarter inches to the left angle of the mouth, and from the border of the mouth to within one-eighth of an inch of the left ala of the nose. You notice this little nodule or tubercle on one side, which illustrates in an excellent way the mode of growth of the epithelial cancer, or carcinoma, showing how actively proliferation goes on.

On last Wednesday I operated on an epithelial cancer of the lower lip in a young man only 35 years of age. Cancer is rare at such an early age. It does not usually attack a person under the age of 50. The treatment in this case, will be, of course, the immediate removal of the growth. The man has been put thoroughly under the influence of ether while I have been speaking to you. The disease, in this instance, does not involve the mucous membrane of the mouth or lips. In cutting, I shall go as far as possible from the limits of disease. This will be a very nasty and bloody operation, and I am much afraid that the man will be pretty thoroughly out of the influence of the ether before I get through with the cutting. I begin by making a rectangular incision down to the bone, cutting well up on the cheek between the cancer and the left ala of the nose, and prolonging the left side of the mouth. Now that I have cut out all the diseased tissue, I proceed to take up the flesh along the canine fossa of the upper jaw-bone, and well up towards the orbit, so that I may slip my flap forward sufficiently. You will always have to expect great hæmorrhage in these operations on the lower parts of the face. Before bringing the flaps together, my assistants carefully tie all these spurting arteries. The hæmorrhage is still considerable, however, for no part of the body is more vascular than the face. By loosening the right side of the upper lip and sliding it well over, you see that I have succeeded admirably in filling up the gap. There may possibly be some slight deformity of the left corner of the mouth, but that will not make much difference, particularly in the case of a man that can let his moustache grow to cover the scar. The mouth looks a little drawn up, but nature will bring it back into shape.

MOTHER'S MARK.—You notice this soft, elastic tumor over the upper portion of the left frontal bone. It is as large as an almond, and is traversed by veins. When the child cries the tumor grows hard and tense. This is what is vulgarly known as a mother's mark, a nævus materna. These tumors are called cavernous angiomæ, and consist of dilated veins, or arteries, or both—sometimes the veins predominate, sometimes the arteries. These veins and arteries are, of course, of capillary size. There are a great many ways of operating in a case like this. In a recent instance I tried to cut away the growth under the skin, so as to avoid a bad looking scar, but I found it of no use. On another occasion, I tried cauterization, heating the bulb of the cautery, and perforating the tumor in many places, but it did no real good. The proper way to treat such cases is the one I shall now adopt. I push two oiled pins right through the base of the growth so that they cross each other at right angles. I then take a sharp knife and cut a grove in the skin between the points of insertion and exit of the two pins, and then pass a stout ligature round the base of the nævus and underneath the pins. I draw this ligature just as tight as I possibly can, so as to completely strangulate the growth. When this is done the vessels of the tumor are obliterated, new matter is thrown out, and the tumor itself sloughs off in the course of four or five days, leaving an open, granulating wound, which must be protected by some mild ointment. Before dismissing the case I cut off the end of the pins, so that they will not catch in the clothing. There is no use whatever in temporizing in these cases by the use of the cautery, or by the injection of irritating substances into the body of the tumor.

SARCOMA OF THE CHEEK.—This man is a farmer, 57 years of age. About one year ago he first noticed a small swelling above the canine fossa of the lower jaw bone. The spot gradually became the seat of lancinating pains. Upon questioning the patient I found that one of his brothers died of cancer of the lower lip. In spite of this local disease, the man's general health during the past year has been excellent; he sleeps well and has a good appetite. You see this irregular, ovoid swelling, extending from the upper lip almost to the orbit, and from the left ala of the nose all the way to the anterior border of the ramus of the lower jaw. This tumor is densely hard and immovably fixed. The mouth does not seem to be involved. The palatine process of the superior maxillary and the alveolus of the jaw bone are free from disease. There is no implication of the orbit. I do not yet know the condition of the nasal bones. When a tumor begins in or over the antrum it usually attacks the jaw in all directions. A cancer of the antrum highmorianum may arise from the glands of the mucous membrane, or from the periosteum. The various tumors occur in the following order, so far as frequency is concerned, viz.: sarcomas, carcinomas, fibromas, cysts and osseous growths. This tumor feels exactly like bone, and is perfectly immovable. Upon introducing my finger into the man's mouth I find that there is no extension of the disease into that cavity. I should say that this tumor originated with the soft layer of the periosteum. It may involve the malar bone. There is evidently incipient ossification in this case. Wherever there is ossification or calcification, we call the tumor osseoid. The osseoid sarcoma is one of the most malignant of all growths. In my late investigations into the malignant tumors of the long bones, I have found that sarcoma is malignant in 75-100 of all the cases. The skin in this case was thickened and disfigured by the application of a "cancer plaster" to the spot by some quack. This of course caused sloughing. I do not find any lymphatic involvement in this case. Malignant disease may exist to a very late period in its course without lymphatic involvement. Now that the patient is thoroughly under the anæsthetic, I shall begin by making a rectangular flap (the skin of the cheek is not at all involved), carrying my knife up along the edge of the nose to a point about an inch below the inner angle of the left eye, and from there well across below the eye towards the ear. I am working my way gradually from the surface towards the antrum. Sarcoma is most common in the young, but is met with at all ages. My first diagnosis of the case was a correct one. It is a case of osseoid sarcoma of the outer layer of the periosteum of the antrum. The bone is no where involved. There is some slight deviation of the septum of the nose, but that is without doubt the result of a severe fracture of that organ, which the man received some time ago. In some rare instances (I do think it is the case here), there is an extension of the disease into the bone through the Haversian canals. I am gradually getting away all the diseased tissue, by means of this chisel. The bone is altogether sound, as are also the muscles. The alveolar process above the first molar is a little soft, but the softness does not extend to any depth. As I find some difficulty in stopping the bleeding from some of the smaller arteries, particularly those issuing from the bone, I shall have to leave the wound open for a while until the flow can be stanched. I will then bring the flaps together by twisted sutures. From present appearances, there will be very good coaptation of the parts.

